# Different K_CO_ and V_A_ combinations exist for the same DL_CO_ value in patients with diffuse parenchymal lung diseases

**DOI:** 10.1186/s12890-015-0084-1

**Published:** 2015-09-03

**Authors:** Jean Pastre, Laurent Plantier, Carole Planes, Raphaël Borie, Hilario Nunes, Christophe Delclaux, Dominique Israël-Biet

**Affiliations:** Université Paris Descartes, Sorbonne Paris Cité and AP-HP, Service de Pneumologie, Hôpital Européen Georges Pompidou, Paris, France; Université Paris Diderot, Sorbonne Paris Cité and AP-HP, Service de Physiologie, Hôpital Bichat-Claude Bernard, Paris, France; Université Paris 13, Sorbonne Paris Cité and AP-HP, Service de Physiologie, Hôpital Avicenne, Bobigny, France; Université Paris Descartes, Sorbonne Paris Cité and AP-HP, Service de Pneumologie, Hôpital Bichat-Claude Bernard, Paris, France; Université Paris 13, Sorbonne Paris Cité and AP-HP, Service de Pneumologie, Hôpital Avicenne, Bobigny, France; Université Paris Descartes, Sorbonne Paris Cité and AP-HP, Service de Physiologie, Hôpital Européen Georges Pompidou, Paris, France

**Keywords:** Carbon monoxide diffusing capacity, DL_CO_, Carbon monoxide transfer coefficient, K_CO_, Interstitial lung disease

## Abstract

**Background:**

DL_CO_ is the product of the CO transfer coefficient (K_CO_) by the “accessible” alveolar volume (V_A_). In theory, the same DL_CO_ may result from various combinations of K_CO_ and V_A_ values, each of which reflect different injury sites and mechanisms. We sought to determine in this study the potential variability of both V_A_ and K_CO_ for fixed values of DL_CO_ in diffuse parenchymal lung diseases (DPLD).

**Methods:**

To this end, we designed a retrospective, cross-sectional study of three distinct types of DPLD and analysed pulmonary function test (PFT) datasets.

**Results:**

We show here that for the same value of DL_CO_ (50 % predicted), K_CO_ varied from 60 to 95 % predicted and V_A_ from 55 to 85 % predicted in various types of DPLD idiopathic pulmonary fibrosis, sarcoidosis and connective tissue disease-associated DPLD, indicating distinct pathogenic mechanisms in these diseases. In addition, a comparison of V_A_ with total lung capacity may help to evidence the distal airway obstruction sometimes associated with certain DPLD particularly sarcoidosis.

**Conclusion:**

Clinicians should take into account not only DL_CO_ but also V_A_ and K_CO_ values when managing patients with DPLD.

## Background

The single-breath carbon monoxide diffusing capacity (DL_CO_) is the product of two measurements during breath holding at full inflation: the rate constant for carbon monoxide uptake from alveolar gas (K_CO_ [minute^−1^]) and the “accessible” alveolar volume (V_A_). Consequently, the same DL_CO_ may result from various combinations of K_CO_ and V_A_ values. Changes in each of K_CO_ and V_A_ may reflect different injury sites and mechanisms. In theory, the decrease in DL_CO_ may result from a fall in V_A_ (mainly due to restrictive and/or obstructive defects) and/or a fall in K_CO_ (due to alveolar/capillary damage or a microvascular disease). Few studies have focused on the significance of DL_CO_ in diffuse parenchymal lung diseases (DPLD) [1–5], highlighting the prognostic value of its component K_CO_. No study to our knowledge has sought to assess the validity of the above mentioned theory in the context of DPLD. Our primary objective in the present study was to assess in a large cohort of distinct types of DPLD the potential variability of both V_A_ and K_CO_ for fixed values of DL_CO_. A secondary objective was to determine whether a low V_A_ value in this context might reflect a distal airway obstruction in addition to a potential restrictive defect. To this end, we designed a retrospective, cross-sectional study of three distinct types of DPLD: idiopathic pulmonary fibrosis (IPF, the prototype for fibrotic pulmonary diseases predominantly affecting the lower lobes), stage IV sarcoidosis (predominantly affecting the upper lobes) and connective tissue disease-associated interstitial lung diseases (CTD-ILDs, which are usually characterized by diffuse, inflammatory lesions rather than fibrotic damage).

## Methods

Each of three university hospitals in France provided pulmonary function test (PFT) datasets from around 80 DPLD patients (75, 80 and 87 patients, respectively). Pulmonary function tests had been performed according to international recommendations and had used similar quality criteria [6–8]. Only raw PFT data were provided and % predicted values were subsequently calculated by a single investigator (CD2) for the whole population according to Stanojevic for spirometry [9] and other international recommendations for DL_CO_ and static lung volumes respectively [10, 11]. The PFTs (spirometry, body plethysmography and single-breath carbon monoxide transfer) using routine techniques had been performed for clinical purposes. We got approval from the Institutional Review Board of the French learned society for respiratory medicine – Société de Pneumologie de Langue française, which judged our study as fully observational and which therefore did not require any informed consent.

Two-hundred and forty-two patients with complete datasets were retrospectively assigned to IPF (n = 85), sarcoidosis (n = 73) or CTD-ILD (n = 84) groups. Patients with IPF and CTD-ILD exhibited lower values of DL_CO_ than those with sarcoidosis (43 ± 18 % predicted (11-89 %), 44 ± 15 (12-88 %), and 56 ± 18 % (19-115 %), in IPF, CTD-ILD and sarcoidosis, respectively, p < 0.0001). Then, three PFT datasets (one per group) were matched for DL_CO_ % predicted (agreement 5 %, by a single investigator (CD2)) to allow comparisons of the groups at similar levels of DL_CO_. Consequently, 77 patients were excluded from the analysis due to matching selection (for instance IPF and CTD-ILD subjects with very low DL_CO_ % predicted values and sarcoidosis subjects with high DL_CO_ values). Results were expressed as means ± SD. Continuous variables were compared using the Student’s *t*-test or the analysis of variance (ANOVA, see Table) as appropriate. The chi-squared test was used for the comparison of qualitative variables (smoking history). Statistical significance was defined by a *p* value <0.05. All analyses were performed using the Statview 4 package (SAS institute, Grenoble, France).

## Results

**Fig. 1:**
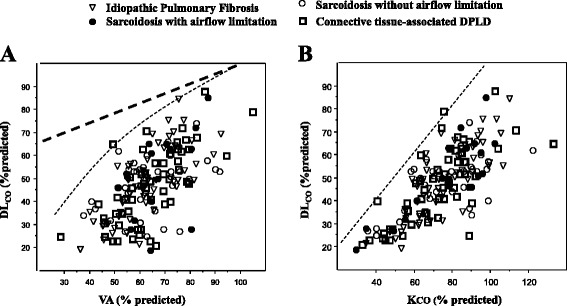
Relationships between DL_CO_ on one hand and V_A_ (left panel) and K_CO_ (right panel) on the other. Circles represent sarcoidosis (closed: with airflow limitation, n = 17; open: without airflow limitation). **a** Dotted lines describe “reduced expansion” (upper bold line) and “loss of units” effects, calculated according to Hughes and Pride [4]. Patients with DPLD lied in the discrete to diffuse loss of alveolar unit areas. **b** The dotted line is the identity line for the DL_CO_-K_CO_ plot; patients along this line have normal V_A_ and the reduced DL_CO_ is related to a decrease in K_CO_ due to microvascular pathology

One hundred and sixty-five PFT datasets (55 per group) were analysed (Table 1). The three study groups had similar mean values for K_CO_ and V_A_ as well as for DL_CO_ (the matching criterion). However, on an individual patient basis, a similar DL_CO_ could be obtained from various combinations of K_CO_ and V_A_ (Fig. 1). This figure clearly shows that K_CO_ can vary from decreased (diffuse loss of units) to normal or barely increased (discrete loss of units) values. We show here that for a similar DL_CO_ value of 50 % predicted, for instance, K_CO_ varied from 60 to 95 % predicted and V_A_ from 55 to 85 % predicted.

**Table 1:** Demographic and functional characteristics of the study participants

	IPF	Sarcoidosis	CTD-ILD	P value (ANOVA)	Between-groups difference
	n = 55	n = 55	n = 55		
	gr. 1	gr. 2	gr. 3		
Centre 1/2/3, n	15/22/18	12/25/18	29/12/14	0.007	Not tested
Gender, F/M	15/40	27/28	24/31	0.048	Not tested
Age, years	71 ± 8	52 ± 11	60 ± 14	<0.001	2<3<1
Height, cm	167 ± 9	168 ± 10	167 ± 9	0.812	
History of smoking	23/27/5	33/19/3	25/26/4	0.383	
(never/ex/current smokers)					
FEV_1_, L	2.17 ± 0.69	1.87 ± 0.65	2.18 ± 0.66	0.023	Not tested
FEV_1_, % predicted	82 ± 21	59 ± 17	74 ± 15	<0.001	2<3<1
FVC, L	2.65 ± 0.68	2.66 ± 0.81	2.65 ± 0.89	0.994	
FVC, % predicted	74 ± 19	66 ± 15	68 ± 15	0.053	
FEV_1_/FVC	0.83 ± 0.07	0.71 ± 0.14	0.84 ± 0.07	<0.001	Not tested
FEV_1_/FVC, % predicted	109 ± 10	90 ± 17	108 ± 9	<0.001	2<1-3
TLC, L	4.50 ± 1.23	4.67 ± 1.20	4.39 ± 1.15	0.486	
TLC, % predicted	75 ± 16	80 ± 17	75 ± 15	0.147	
FRC, L	2.51 ± 0.69	2.65 ± 0.64	2.53 ± 0.70	0.582	
FRC, % predicted	77 ± 18	87 ± 25	81 ± 20	0.038	1<2
RV, L	1.76 ± 0.47	1.90 ± 0.69	1.67 ± 0.41	0.078	
RV, % predicted	73 ± 18	97 ± 30	80 ± 22	<0.001	1-3<2
RV/TLC	0.40 ± 0.06	0.41 ± 0.09	0.39 ± 0.07	0.362	
V_A_, L	3.66 ± 0.96	3.70 ± 0.92	3.66 ± 1.01	0.972	
K_CO_, mmol/min/kPa/L	1.00 ± 0.23	1.20 ± 0.30	1.07 ± 0.30	<0.001	
K_CO_, % predicted	75 ± 17	77 ± 20	72 ± 19	0.507	Not tested
DL_CO_, mmol/min/kPa	3.68 ± 1.37	4.45 ± 1.65	4.02 ± 1.66	0.040	
DL_CO_, % predicted	**48 ± 15**	**49 ± 14**	**47 ± 16**	**0.737**	Not tested
V_A_/TLC	0.81 ± 0.06	0.80 ± 0.08	0.83 ± 0.06	0.047	3>2

Abbreviations: *IPF* idiopathic pulmonary fibrosis, *CTD-ILDs* connective tissue disease-associated interstitial lung diseases, *FVC* forced vital capacity, *FEV*_*1*_ forced expiratory volume in 1 s, *FRC* forced respiratory capacity, *TLC* total lung capacity, *DL*_*CO*_ carbon monoxide diffusing capacity, *K*_*CO*_ rate for carbon monoxide uptake, *V*_*A*_ alveolar volume

In addition, 17 patients exhibited an airflow limitation (FEV_1_/FVC < lower limit of normal). They all belonged to the sarcoidosis group (Table 1). The reduction in alveolar volume (measured using a dilution technique) relative to total lung volume (TLC, measured using body plethysmography), expressed as V_A_/TLC, was correlated with parameters of central airway obstruction (FEV_1_/FVC: r^2^ = 0.10, p < 0.001) and even more strongly with distal airway obstruction (RV/TLC: r^2^ = 0.25, p < 0.001). Since the V_A_/TLC value of the population as a whole may seem lower than expected (Table 1) even in patients without significant airflow limitation (n = 148, FEV_1_/FVC = 0.82 ± 0.06), we further evaluated whether some patients exhibited a small airways obstructive syndrome defined by a normal FEV_1_/FVC ratio and a greater reduction of both FEV_1_ and FVC than TLC (FVC % predicted/TLC % predicted < 0.80). We found 20 such subjects, described in Table 2. Similarly to proximal airflow limitation, small airways obstructive syndrome was predominantly present in sarcoidosis.

**Table 2:** Small airway obstructive syndrome (SAOS) in patients without proximal airflow limitation (FEV1/FVC > lower limit of normal)

Characteristic	With SAOS	Without SAOS	P value
	N = 20	N = 128	
IPF/sarcoidosis/CTD-ILD, n	2/11/7	53/27/48	0.002
Gender, F/M	14/6	45/83	0.006
Age, years	54 ± 14	64 ± 13	0.003
Body mass index, kg.m^−2^	25.8 ± 5.3	26.2 ± 3.8	0.664
FEV_1_, % predicted	55 ± 13	78 ± 17	<0.001
FVC, % predicted	54 ± 14	72 ± 16	<0.001
FEV_1_/FVC, % predicted	101 ± 13	107 ± 10	0.031
TLC, % predicted	75 ± 17	76 ± 15	0.786
FRC, % predicted	83 ± 23	78 ± 19	0.309
RV, % predicted	98 ± 27	76 ± 19	<0.001
RV/TLC	0.48 ± 0.07	0.38 ± 0.06	<0.001
V_A_/TLC	0.77 ± 0.07	0.83 ± 0.05	<0.001

Abbreviations: *IPF* idiopathic pulmonary fibrosis, *CTD-ILDs* connective tissue disease-associated interstitial lung diseases, *FVC* forced vital capacity, *FEV*_*1*_ forced expiratory volume in 1 s, *FRC* forced respiratory capacity, *TLC* total lung capacity, *V*_*A*_ alveolar volume

## Discussion

Our present study confirms that an abnormally low DL_CO_ can result from very different combinations of the primary measurements K_CO_ and V_A_. This was the case for all three types of DPLD. Furthermore, the assessment of V_A_/TLC [12], the latter being obtained from body plethysmography, may suggest both central or peripheral airway obstruction and this was observed particularly in sarcoidosis thereby providing additional clues to the pathogenic features of this condition. We recently described diseases associated with a small airway obstructive syndrome (a non-specific pattern frequently observed in pulmonary function testing units [13]). It is noteworthy that in that study, sarcoidosis and interstitial pneumonia were two of the conditions associated with this pattern. In the present work, we extend our previous data showing that a DPLD can exhibit a mixed pattern associating both a restrictive syndrome and a small airways obstructive syndrome.

## Conclusions

In conclusion, we confirmed that the components of DL_CO_ (K_CO_ and V_A_) may largely vary in DPLD while DL_CO_ appears constant. The magnitudes of K_CO_ and V_A_ values might indicate distinct disease mechanisms and thereby bear a relative prognostic value in addition to giving clues to pathogenesis of these diseases. For these reasons, clinicians should take into account not only DL_CO_ but also V_A_ and K_CO_ when seeking to assess DPLD, in order to provide a more informed and better care to these patients.

## Abbreviations

DL_CO_: carbon monoxide diffusing capacity
K_CO_: rate for carbon monoxide uptake
V_A_: alveolar volume
DPLD: diffuse parenchymal lung disease
IPF: idiopathic pulmonary fibrosis
CTD-ILDs: connective tissue disease-associated interstitial lung diseases
PFT: pulmonary function test
SDS: standard deviation score
FVC: forced vital capacity
FEV_1_: forced expiratory volume in 1 second
FRC: forced respiratory capacity
TLC: total lung capacity
sRaw: specific airway resistance
